# Evaluation of a fiberoptic-based system for measurement of optical properties in highly attenuating turbid media

**DOI:** 10.1186/1475-925X-5-49

**Published:** 2006-08-23

**Authors:** Divyesh Sharma, Anant Agrawal, L Stephanie Matchette, T Joshua Pfefer

**Affiliations:** 1 Food and Drug Administration, Center for Devices and Radiological Health, Rockville, Maryland, USA

## Abstract

**Background:**

Accurate measurements of the optical properties of biological tissue in the ultraviolet A and short visible wavelengths are needed to achieve a quantitative understanding of novel optical diagnostic devices. Currently, there is minimal information on optical property measurement approaches that are appropriate for *in vivo *measurements in highly absorbing and scattering tissues. We describe a novel fiberoptic-based reflectance system for measurement of optical properties in highly attenuating turbid media and provide an extensive *in vitro *evaluation of its accuracy. The influence of collecting reflectance at the illumination fiber on estimation accuracy is also investigated.

**Methods:**

A neural network algorithm and reflectance distributions from Monte Carlo simulations were used to generate predictive models based on the two geometries. Absolute measurements of diffuse reflectance were enabled through calibration of the reflectance system. Spatially-resolved reflectance distributions were measured in tissue phantoms at 405 nm for absorption coefficients (μ_a_) from 1 to 25 cm^-1 ^and reduced scattering coefficients (μ′s
 MathType@MTEF@5@5@+=feaafiart1ev1aaatCvAUfKttLearuWrP9MDH5MBPbIqV92AaeXatLxBI9gBaebbnrfifHhDYfgasaacH8akY=wiFfYdH8Gipec8Eeeu0xXdbba9frFj0=OqFfea0dXdd9vqai=hGuQ8kuc9pgc9s8qqaq=dirpe0xb9q8qiLsFr0=vr0=vr0dc8meaabaqaciaacaGaaeqabaqabeGadaaakeaacuaH8oqBgaqbamaaBaaaleaacqqGZbWCaeqaaaaa@3007@) from 5 to 25 cm^-1^. These data and predictive models were used to estimate the optical properties of tissue-simulating phantoms.

**Results:**

By comparing predicted and known optical properties, the average errors for μ_a _and μ′s
 MathType@MTEF@5@5@+=feaafiart1ev1aaatCvAUfKttLearuWrP9MDH5MBPbIqV92AaeXatLxBI9gBaebbnrfifHhDYfgasaacH8akY=wiFfYdH8Gipec8Eeeu0xXdbba9frFj0=OqFfea0dXdd9vqai=hGuQ8kuc9pgc9s8qqaq=dirpe0xb9q8qiLsFr0=vr0=vr0dc8meaabaqaciaacaGaaeqabaqabeGadaaakeaacuaH8oqBgaqbamaaBaaaleaacqqGZbWCaeqaaaaa@3007@ were found to be 3.0% and 4.6%, respectively, for a linear probe approach. When bifurcated probe data was included and samples with μ_a _values less than 5 cm^-1 ^were excluded, predictive errors for μ_a _and μ′s
 MathType@MTEF@5@5@+=feaafiart1ev1aaatCvAUfKttLearuWrP9MDH5MBPbIqV92AaeXatLxBI9gBaebbnrfifHhDYfgasaacH8akY=wiFfYdH8Gipec8Eeeu0xXdbba9frFj0=OqFfea0dXdd9vqai=hGuQ8kuc9pgc9s8qqaq=dirpe0xb9q8qiLsFr0=vr0=vr0dc8meaabaqaciaacaGaaeqabaqabeGadaaakeaacuaH8oqBgaqbamaaBaaaleaacqqGZbWCaeqaaaaa@3007@ were further reduced to 1.8% and 3.5%.

**Conclusion:**

Improvements in system design have led to significant reductions in optical property estimation error. While the incorporation of a bifurcated illumination fiber shows promise for improving the accuracy of μ′s
 MathType@MTEF@5@5@+=feaafiart1ev1aaatCvAUfKttLearuWrP9MDH5MBPbIqV92AaeXatLxBI9gBaebbnrfifHhDYfgasaacH8akY=wiFfYdH8Gipec8Eeeu0xXdbba9frFj0=OqFfea0dXdd9vqai=hGuQ8kuc9pgc9s8qqaq=dirpe0xb9q8qiLsFr0=vr0=vr0dc8meaabaqaciaacaGaaeqabaqabeGadaaakeaacuaH8oqBgaqbamaaBaaaleaacqqGZbWCaeqaaaaa@3007@ estimates, further study of this approach is needed to elucidate the source of discrepancies between measurements and simulation results at low μ_a _values.

## Background

In recent years, advances in optical technology have helped facilitate rapid progress in fluorescence and reflectance spectroscopy-based techniques for medical diagnostics. Recent studies involving ultraviolet A (UVA, 320 to 400 nm) and shorter visible (VIS) wavelengths (400 to 550 nm) have demonstrated that spectroscopic approaches can be highly effective for a variety of applications including intravascular detection of atherosclerotic plaque [1], *in situ *brain tumor demarcation [2] and surveillance for neoplasia in mucosal tissues that line organs such as the lungs and cervix [3]. While clinical studies have shown significant promise, further improvements in efficacy are needed for this technology to achieve its full potential.

Accurate approaches for *in vivo *measurement of tissue optical properties in UVA and short VIS wavelengths are needed to optimize the ability of diagnostic devices. Since some tissue discrimination algorithms [4] use optical property data as inputs, the accuracy of this data may directly influence a system's diagnostic efficacy. Furthermore, numerical and analytical models are effective tools for elucidating light-tissue interaction phenomena and identifying optimal device designs, including selection of excitation and collection wavelengths and probe geometry [5,6]. However, the accuracy of input parameters such as tissue optical properties can strongly influence the quality and usefulness of modeling results.

The most important optical properties for describing light propagation in tissue are absorption coefficient (μ_a_) and reduced scattering coefficient {μ′s
 MathType@MTEF@5@5@+=feaafiart1ev1aaatCvAUfKttLearuWrP9MDH5MBPbIqV92AaeXatLxBI9gBaebbnrfifHhDYfgasaacH8akY=wiFfYdH8Gipec8Eeeu0xXdbba9frFj0=OqFfea0dXdd9vqai=hGuQ8kuc9pgc9s8qqaq=dirpe0xb9q8qiLsFr0=vr0=vr0dc8meaabaqaciaacaGaaeqabaqabeGadaaakeaacuaH8oqBgaqbamaaBaaaleaacqqGZbWCaeqaaaaa@3007@ = μ_s_(1-g)}, which is a convenient combination of the scattering coefficient (μ_s_) and the scattering phase function (g). While prior optical property studies have typically involved VIS to near-infrared wavelengths where tissue tends to have relatively low attenuation levels [7], data on *in vivo *optical properties in the UVA and lower VIS wavelength ranges are limited. This is likely due in part to the fact that many earlier studies were performed in support of photon migration applications. Another factor may have been the difficulties associated with measuring signals in tissue with high attenuation levels, such as the rapid decay in reflected light levels with distance from the source location.

In spite of the limited literature on optical property measurements at relevant UVA and short VIS wavelengths, prior studies at longer wavelengths have produced significant advances in experimental, computational and analytical techniques, some of which are applicable in our spectral range of interest. While time-[8] and frequency-[9] domain approaches have shown promise at long VIS and near-infrared wavelengths, these approaches tend to be expensive and have not shown efficacy at shorter wavelengths. Spatially-resolved approaches typically involve using tissue phantoms or computer simulations to generate data for inverse model calibration, and then using the model to estimate optical properties based on reflectance measurements [10]. In prior studies, diffuse reflectance has been collected from tissue phantoms over a broad range of optical properties and wavelengths [10-12]. Monte Carlo [10,11] or diffusion approximation [13] light propagation models are typically used to generate spatially-resolved reflectance profiles, although the latter are limited to larger source-detector separation distances which satisfy diffusion criteria. Reflectance measurements are typically performed with multiple-channel fiberoptic bundles, or probes, which deliver light to the tissue surface and collect reflectance at two or more well-defined distances from the source fiber. Optical fibers are commonly arranged in either circular [11-14] or linear [15,16] patterns on the face of the fiberoptic probe. The source-collection separation distances used in prior studies have ranged from several millimeters to centimeters [11,14-18]. Properties such as numerical aperture (NA) and fiber diameter have varied widely in prior studies. One recent study involved an approach based on bifurcated probes of varying aperture diameter, rather than the more typical linear array approach [19]. Multivariate calibration techniques such as neural networks (NN) [10,13,20], fuzzy logic [21], regression [11] and partial least squares [22] have been used to solve the inverse problem of determining optical properties from reflectance.

In a prior pilot study, we evaluated several different approaches to fiberoptic probe-based optical property determination using Monte Carlo simulations and diffuse reflectance measurements [23]. By calibrating a neural network model with simulated reflectance data based on uniformly distributed optical properties and validating the model with measurements of tissue phantoms having randomly distributed optical properties, moderately high levels of accuracy were found: root mean square errors of 1.58 cm^-1 ^for μ_a _and 2.35 cm^-1 ^for μ′s
 MathType@MTEF@5@5@+=feaafiart1ev1aaatCvAUfKttLearuWrP9MDH5MBPbIqV92AaeXatLxBI9gBaebbnrfifHhDYfgasaacH8akY=wiFfYdH8Gipec8Eeeu0xXdbba9frFj0=OqFfea0dXdd9vqai=hGuQ8kuc9pgc9s8qqaq=dirpe0xb9q8qiLsFr0=vr0=vr0dc8meaabaqaciaacaGaaeqabaqabeGadaaakeaacuaH8oqBgaqbamaaBaaaleaacqqGZbWCaeqaaaaa@3007@. These values translate to average predictive errors of 12.5% and 16.2%. Optical property estimation errors may be attributed in part to the measurement approach in which large variations in intensity from fiber-to-fiber were produced at the CCD camera, as well as the limited dynamic range (14-bit) and noise levels generated during long exposures. In general, these results were sufficient for a pilot study, yet they indicated that refinements would be needed before accurate *in vivo *measurements could be achieved.

In an effort to improve upon the results of the pilot study and develop a system that is sufficiently accurate to provide meaningful data during *in vivo *measurements, significant modifications were made to the prior system. The goals of the current study were to evaluate the accuracy of this second generation optical property measurement system and assess the potential added benefit of a bifurcated fiberoptic probe that enables detection of reflectance from the illumination site.

## Methods

### System description

**Figure 1:**
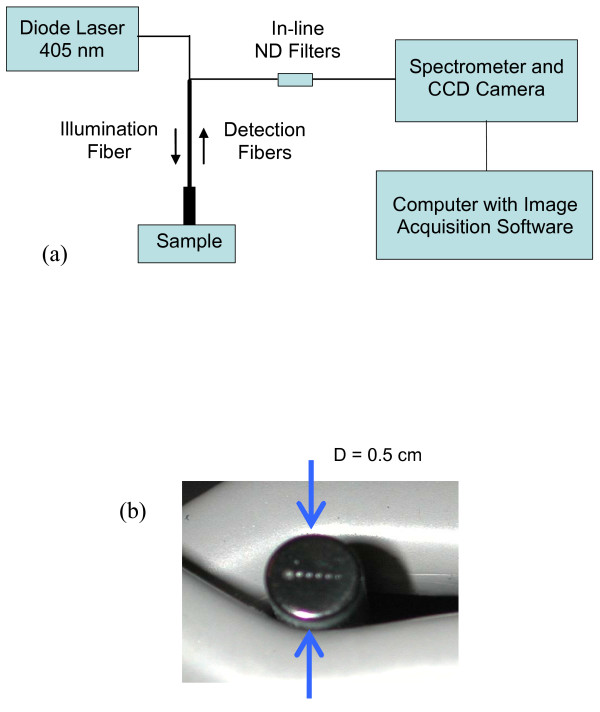
**System Diagram**. Diagram includes (a) experimental setup and (b) fiberoptic probe face.

Diagrams of the experimental setup and fiberoptic probe face used to perform diffuse reflectance measurements are presented in Figure 1. The source is a 405 nm diode laser (LCDU 12/5431, Power Technology, Inc; Little Rock, AR) with a power level of 2.5 mW. The input power is adjusted using neutral density (ND) filters (CVI Laser Corporation, Albuquerque, NM). The custom designed fiberoptic probe (FiberTech Optica, Ontario, Canada) is used to deliver laser light from the source to the sample and guide diffuse reflectance from the sample to the detector. The diameter of the probe face is 0.5 cm which would make it practical for *in vivo *studies of internal organ sites. The probe contains a single illumination fiber and five detection fibers spaced at consecutive center-to-center distances of 0.5 mm. The core diameter of each fiber is 0.2 mm with a NA of 0.22. The detection legs are connected to five in-line filters (FHS-UV, Ocean Optics, Dunedin, FL) three of which contained ND filters. The filter holders are coupled to the legs of a second probe, the common end of which contains a linear fiber array that was aligned to the entrance slit of an imaging spectrometer (SpectraPro 300i, Acton Research Corp., Acton, MA). The output of the spectrometer is detected by a low noise, high dynamic range (16-bit) CCD camera (Princeton Instruments Spec10:400B, Trenton, New Jersey) and acquired using proprietary software (WinSpec Princeton Instruments, Trenton, NJ).

### Experimental system modifications

**Figure 2:**
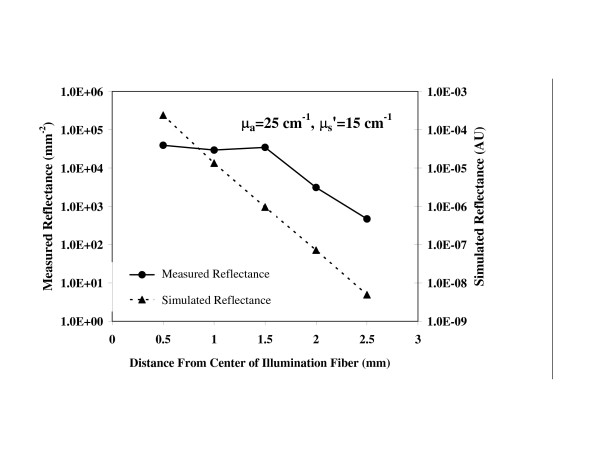
**Reduction of detected signal range**. Monte Carlo simulations and experimental data for μ_a _= 25 cm^-1^, μ′s
 MathType@MTEF@5@5@+=feaafiart1ev1aaatCvAUfKttLearuWrP9MDH5MBPbIqV92AaeXatLxBI9gBaebbnrfifHhDYfgasaacH8akY=wiFfYdH8Gipec8Eeeu0xXdbba9frFj0=OqFfea0dXdd9vqai=hGuQ8kuc9pgc9s8qqaq=dirpe0xb9q8qiLsFr0=vr0=vr0dc8meaabaqaciaacaGaaeqabaqabeGadaaakeaacuaH8oqBgaqbamaaBaaaleaacqqGZbWCaeqaaaaa@3007@ = 15 cm^-1^. The signal range in the experimental data was reduced by using neutral density filters to preferentially attenuate the channels closest to the source fiber.

In our previous system, the large range of light levels (Figure 2) incident on the detector necessitated measurements at multiple exposure durations to acquire a single reflectance distribution. By improving the homogeneity of light levels delivered by the fiberoptic probe, a single CCD acquisition could record all fiber intensities. This was achieved by using in-line ND filters [24] for each fiber. By analyzing the detected signal levels for four samples at the edges of the designated optical property parameter space (μ_a _= 1, 25 cm^-1^, μ′s
 MathType@MTEF@5@5@+=feaafiart1ev1aaatCvAUfKttLearuWrP9MDH5MBPbIqV92AaeXatLxBI9gBaebbnrfifHhDYfgasaacH8akY=wiFfYdH8Gipec8Eeeu0xXdbba9frFj0=OqFfea0dXdd9vqai=hGuQ8kuc9pgc9s8qqaq=dirpe0xb9q8qiLsFr0=vr0=vr0dc8meaabaqaciaacaGaaeqabaqabeGadaaakeaacuaH8oqBgaqbamaaBaaaleaacqqGZbWCaeqaaaaa@3007@ = 5, 25 cm^-1^) we identified a combination of ND filters for each fiber which would produce moderately high output levels on the CCD without inducing saturation. Combined attenuation levels of 2.2, 1.9, and 0.3 OD were used for the fibers at separation distances of 0.5, 1.0 and 2.0 mm. The ND levels chosen for these fibers appear irregular due to variations in fiber transmittance and in coupling efficiency of the in-line filters. Thus the system was optimized in such a way that a single set of ND filters could be used to collect reflectance for *in vitro *or *in vivo *samples having optical properties anywhere within the relevant range (μ_a _= 1–25 cm^-1^, μ′s
 MathType@MTEF@5@5@+=feaafiart1ev1aaatCvAUfKttLearuWrP9MDH5MBPbIqV92AaeXatLxBI9gBaebbnrfifHhDYfgasaacH8akY=wiFfYdH8Gipec8Eeeu0xXdbba9frFj0=OqFfea0dXdd9vqai=hGuQ8kuc9pgc9s8qqaq=dirpe0xb9q8qiLsFr0=vr0=vr0dc8meaabaqaciaacaGaaeqabaqabeGadaaakeaacuaH8oqBgaqbamaaBaaaleaacqqGZbWCaeqaaaaa@3007@ = 5–25 cm^-1^). Figure 2 shows computational modeling results for a sample with μ_a _= 25 cm^-1^, μ′s
 MathType@MTEF@5@5@+=feaafiart1ev1aaatCvAUfKttLearuWrP9MDH5MBPbIqV92AaeXatLxBI9gBaebbnrfifHhDYfgasaacH8akY=wiFfYdH8Gipec8Eeeu0xXdbba9frFj0=OqFfea0dXdd9vqai=hGuQ8kuc9pgc9s8qqaq=dirpe0xb9q8qiLsFr0=vr0=vr0dc8meaabaqaciaacaGaaeqabaqabeGadaaakeaacuaH8oqBgaqbamaaBaaaleaacqqGZbWCaeqaaaaa@3007@ = 15 cm^-1 ^in order to illustrate the wide range of intensities that must be detected. This graph also includes a measurement of a tissue phantom with the same optical properties, indicating that the variation of intensity at the CCD has been reduced from five orders of magnitude to two through the use of ND filters. The exposure duration required for acquiring sufficient signal ranged from 0.12 to 25 seconds depending on the sample attenuation level.

The second optimization task involved enabling absolute measurements by calibrating the measured intensity to simulation results. In order to accomplish this goal, CCD measurements for all detection fibers were made at two different illumination intensities in three different phantoms : μ_a _= 1 cm^-1 ^and μ′s
 MathType@MTEF@5@5@+=feaafiart1ev1aaatCvAUfKttLearuWrP9MDH5MBPbIqV92AaeXatLxBI9gBaebbnrfifHhDYfgasaacH8akY=wiFfYdH8Gipec8Eeeu0xXdbba9frFj0=OqFfea0dXdd9vqai=hGuQ8kuc9pgc9s8qqaq=dirpe0xb9q8qiLsFr0=vr0=vr0dc8meaabaqaciaacaGaaeqabaqabeGadaaakeaacuaH8oqBgaqbamaaBaaaleaacqqGZbWCaeqaaaaa@3007@ = 5 cm^-1^, μ_a _= 1 cm^-1 ^and μ′s
 MathType@MTEF@5@5@+=feaafiart1ev1aaatCvAUfKttLearuWrP9MDH5MBPbIqV92AaeXatLxBI9gBaebbnrfifHhDYfgasaacH8akY=wiFfYdH8Gipec8Eeeu0xXdbba9frFj0=OqFfea0dXdd9vqai=hGuQ8kuc9pgc9s8qqaq=dirpe0xb9q8qiLsFr0=vr0=vr0dc8meaabaqaciaacaGaaeqabaqabeGadaaakeaacuaH8oqBgaqbamaaBaaaleaacqqGZbWCaeqaaaaa@3007@ = 25 cm^-1 ^and μ_a _= 2 cm^-1 ^and μ′s
 MathType@MTEF@5@5@+=feaafiart1ev1aaatCvAUfKttLearuWrP9MDH5MBPbIqV92AaeXatLxBI9gBaebbnrfifHhDYfgasaacH8akY=wiFfYdH8Gipec8Eeeu0xXdbba9frFj0=OqFfea0dXdd9vqai=hGuQ8kuc9pgc9s8qqaq=dirpe0xb9q8qiLsFr0=vr0=vr0dc8meaabaqaciaacaGaaeqabaqabeGadaaakeaacuaH8oqBgaqbamaaBaaaleaacqqGZbWCaeqaaaaa@3007@ = 25 cm^-1^. For each fiber position, the relationship between measured CCD counts and Monte Carlo-predicted intensity levels were graphed for the two phantoms. A linear fit to these points and the origin was calculated for each fiber. These linear fits were used as calibration equations to convert CCD counts to absolute intensity levels for all measurements during this study.

### Bifurcated fiberoptic probe measurements

**Figure 3:**
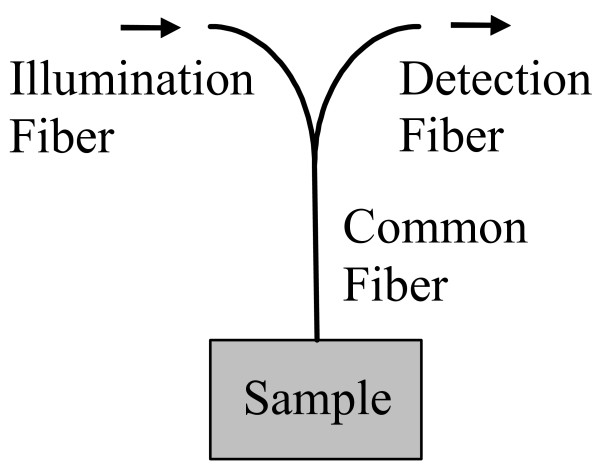
**Diagram of bifurcated probe**. A bifurcated fiberoptic probe was used to measure reflectance at the illumination site.

The objective of this component of the study was to evaluate the utility of adding a bifurcated illumination fiber to the linear-array geometry. This work was performed for two primary reasons: (1) computational results that indicate that at positions near the illumination fiber there is a relatively high level of variation in reflectance intensity as μ′s
 MathType@MTEF@5@5@+=feaafiart1ev1aaatCvAUfKttLearuWrP9MDH5MBPbIqV92AaeXatLxBI9gBaebbnrfifHhDYfgasaacH8akY=wiFfYdH8Gipec8Eeeu0xXdbba9frFj0=OqFfea0dXdd9vqai=hGuQ8kuc9pgc9s8qqaq=dirpe0xb9q8qiLsFr0=vr0=vr0dc8meaabaqaciaacaGaaeqabaqabeGadaaakeaacuaH8oqBgaqbamaaBaaaleaacqqGZbWCaeqaaaaa@3007@ changes [23]; and (2) a prior investigation has indicated that a sized-fiber approach involving collecting reflectance at the site of illumination is highly effective for determining tissue optical properties [19]. A bifurcated fiberoptic probe (Innova Quartz, Phoenix, AZ) – illumination and collection fibers fused to a third common fiber which contacted the sample – with a core diameter of 0.2 mm and NA of 0.22 was used to perform the measurements (Figure 3) [25]. Light collected from the detection leg of the probe was measured by a power meter (Newport Model 1930C, 818-ST-UV detector, Irvine, CA) for each of the 60 samples previously used for the linear array measurements.

The reflectance collected at the illumination site of the sample is the total reflectance (R_total_) which consists of two components: diffuse reflectance (R_diffuse_) and specular reflectance (R_specular_). Fresnel reflections for both fiber core/air and fiber core/water were measured. The probe geometry resulted in multiple reflections at the bifurcation point. Thus, baseline reflectance measurement was made using fiber core refractive index matching liquid (n = 1.47, Cargille Laboratories, Inc; Cedar Grove, NJ). R_diffuse _was obtained by normalizing all the data points to the Fresnel reflections for the fiber core/water as shown below:

*R*_*diffuse *_= *R*_*total *_- *R*_*specular *_    (1)

Rtotal=(Psample−Pη)E.F.∗Pinput     (2)
 MathType@MTEF@5@5@+=feaafiart1ev1aaatCvAUfKttLearuWrP9MDH5MBPbIqV92AaeXatLxBI9gBaebbnrfifHhDYfgasaacH8akY=wiFfYdH8Gipec8Eeeu0xXdbba9frFj0=OqFfea0dXdd9vqai=hGuQ8kuc9pgc9s8qqaq=dirpe0xb9q8qiLsFr0=vr0=vr0dc8meaabaqaciaacaGaaeqabaqabeGadaaakeaacqWGsbGudaWgaaWcbaGaemiDaqNaem4Ba8MaemiDaqNaemyyaeMaemiBaWgabeaakiabg2da9maalaaabaWaaeWaceaacqWGqbaudaWgaaWcbaGaem4CamNaemyyaeMaemyBa0MaemiCaaNaemiBaWMaemyzaugabeaakiabgkHiTiabdcfaqnaaBaaaleaaiiGacqWF3oaAaeqaaaGccaGLOaGaayzkaaaabaGaemyrauKaeiOla4IaemOrayKaeiOla4Iaey4fIOIaemiuaa1aaSbaaSqaaiabdMgaPjabd6gaUjabdchaWjabdwha1jabdsha0bqabaaaaOGaaCzcaiaaxMaadaqadiqaaiabikdaYaGaayjkaiaawMcaaaaa@5649@

Rspecular=(Pwater−Pη)E.F.∗Pinput     (3)
 MathType@MTEF@5@5@+=feaafiart1ev1aaatCvAUfKttLearuWrP9MDH5MBPbIqV92AaeXatLxBI9gBaebbnrfifHhDYfgasaacH8akY=wiFfYdH8Gipec8Eeeu0xXdbba9frFj0=OqFfea0dXdd9vqai=hGuQ8kuc9pgc9s8qqaq=dirpe0xb9q8qiLsFr0=vr0=vr0dc8meaabaqaciaacaGaaeqabaqabeGadaaakeaacqWGsbGudaWgaaWcbaGaem4CamNaemiCaaNaemyzauMaem4yamMaemyDauNaemiBaWMaemyyaeMaemOCaihabeaakiabg2da9maalaaabaWaaeWaceaacqWGqbaudaWgaaWcbaGaem4DaCNaemyyaeMaemiDaqNaemyzauMaemOCaihabeaakiabgkHiTiabdcfaqnaaBaaaleaaiiGacqWF3oaAaeqaaaGccaGLOaGaayzkaaaabaGaemyrauKaeiOla4IaemOrayKaeiOla4Iaey4fIOIaemiuaa1aaSbaaSqaaiabdMgaPjabd6gaUjabdchaWjabdwha1jabdsha0bqabaaaaOGaaCzcaiaaxMaadaqadiqaaiabiodaZaGaayjkaiaawMcaaaaa@5915@

Where,

*P*_*sample *_= Power measured from the sample

*P*_*η *_= Power measured from the index matching liquid

*P*_*water *_= Power output from fiber core/water

*P*_*input *_= Power output from the probe

*E.F*. = Fiber efficiency factor obtained for the collection fiber

Experimentally measured Fresnel reflectance was within 10% of the theoretical value for the fiber core/water interface. The fiber efficiency factor was obtained by coupling light from a 100 μm core diameter fiber into the common end of the bifurcated fiber and measuring the output from the detection leg of the probe. Measurements were made on the same sets of phantoms used for linear-array fiber system.

### Tissue phantoms

Since the validity of the system evaluation is dependent on the accuracy of tissue phantom optical properties, evaluation of phantom materials and benchmarking of phantoms was performed at the outset of this study. The optical properties of individual tissue phantoms were determined using an inverse adding-doubling approach [26] and a spectrophotometer (Shimadzu UV-3101PC, Columbia, MD). This data was compared to theoretical estimates based on Mie theory and direct collimated absorption measurements of the scatterer and absorber, respectively.

Polystyrene microspheres have been used to provide scattering for tissue phantoms in prior studies due to their minimal levels of fluorescence and absorbance as well as their ability to remain in suspension for a long durations. Furthermore, microspheres have a g value which is close to that of biological tissue [27,28]. In order to achieve a g of approximately 0.9, microspheres with a particle size of 1.053 μm (and thus a g of 0.912) were chosen for this study. In order to insure that the absorber used was a pure absorber, absorption and thus transmission should be linear with the concentration in the desired range of absorption coefficient. These measurements were validated against an independent laser setup. As observed in a prior study [29], India ink is particulate in nature with a significant scattering component. Thus, Nigrosin which is the most common absorber cited in literature and which has a linear transmittance with concentration, was chosen as the absorber. The stock absorption coefficient was measured in a spectrophotometer and subsequently diluted to get the required μ_a _for the phantom within the range 1 to 25 cm^-1^.

For the reflectance study, a set of 10 phantoms with uniform optical properties and a set of 60 phantoms with random optical properties were constructed. All phantoms were in liquid form, which enabled us to slightly submerge the tip of the fiberoptic probe for optimal fiber-sample coupling. The uniform optical properties had the following values: 1) with μ_a _held constant at 15 cm^-1 ^and μ′s
 MathType@MTEF@5@5@+=feaafiart1ev1aaatCvAUfKttLearuWrP9MDH5MBPbIqV92AaeXatLxBI9gBaebbnrfifHhDYfgasaacH8akY=wiFfYdH8Gipec8Eeeu0xXdbba9frFj0=OqFfea0dXdd9vqai=hGuQ8kuc9pgc9s8qqaq=dirpe0xb9q8qiLsFr0=vr0=vr0dc8meaabaqaciaacaGaaeqabaqabeGadaaakeaacuaH8oqBgaqbamaaBaaaleaacqqGZbWCaeqaaaaa@3007@ = 5, 10, 15, 20 and 25 cm^-1^; and, 2) with μ′s
 MathType@MTEF@5@5@+=feaafiart1ev1aaatCvAUfKttLearuWrP9MDH5MBPbIqV92AaeXatLxBI9gBaebbnrfifHhDYfgasaacH8akY=wiFfYdH8Gipec8Eeeu0xXdbba9frFj0=OqFfea0dXdd9vqai=hGuQ8kuc9pgc9s8qqaq=dirpe0xb9q8qiLsFr0=vr0=vr0dc8meaabaqaciaacaGaaeqabaqabeGadaaakeaacuaH8oqBgaqbamaaBaaaleaacqqGZbWCaeqaaaaa@3007@ held constant at 15 cm^-1 ^and μ_a _= 1, 5, 10, 15, 20 and 25 cm^-1^. The random optical properties were distributed over a μ_a _range of 1 to 25 cm^-1 ^and μ′s
 MathType@MTEF@5@5@+=feaafiart1ev1aaatCvAUfKttLearuWrP9MDH5MBPbIqV92AaeXatLxBI9gBaebbnrfifHhDYfgasaacH8akY=wiFfYdH8Gipec8Eeeu0xXdbba9frFj0=OqFfea0dXdd9vqai=hGuQ8kuc9pgc9s8qqaq=dirpe0xb9q8qiLsFr0=vr0=vr0dc8meaabaqaciaacaGaaeqabaqabeGadaaakeaacuaH8oqBgaqbamaaBaaaleaacqqGZbWCaeqaaaaa@3007@ range of 5 to 25 cm^-1^.

### Neural network modeling

Evaluation of the optical property measurement technique was performed by developing a NN based inverse model [23] which when provided reflectance data collected from a sample will generate an estimation of μ_a _and μ′s
 MathType@MTEF@5@5@+=feaafiart1ev1aaatCvAUfKttLearuWrP9MDH5MBPbIqV92AaeXatLxBI9gBaebbnrfifHhDYfgasaacH8akY=wiFfYdH8Gipec8Eeeu0xXdbba9frFj0=OqFfea0dXdd9vqai=hGuQ8kuc9pgc9s8qqaq=dirpe0xb9q8qiLsFr0=vr0=vr0dc8meaabaqaciaacaGaaeqabaqabeGadaaakeaacuaH8oqBgaqbamaaBaaaleaacqqGZbWCaeqaaaaa@3007@. The NN algorithm implemented a feed-forward backpropagation network with a Levenburg-Marquardt training function. The input layer contained five and six nodes for the linear array and bifurcated designs, respectively. The output layer contained two nodes, for the predicted optical properties. Optical property calculations were performed offline and required minimal processing time (< 1 sec) for data files that incorporated as many as 60 samples.

Raw reflectance data from computational or experimental results for linear-array probe were preprocessed prior to use in the neural network model:

*S *= -log *R *    (4)

where *R *is the power of the detected reflectance normalized to the illumination power. NN model calibration was performed using 30 sets of simulated-uniform reflectance (*S*) distributions, generated by the Monte Carlo modeling approach described previously [23,30]. Validation was performed against the 30 simulated-uniform (self-validation) and 60 measured phantoms with randomly distributed optical properties.

The linear-array probe data used to calibrate and test the NN models contained reflectance sets comprised of five S values, corresponding to the five detection fibers. When evaluating the models for the bifurcated probe system, the sixth unprocessed (no log taken) reflectance value – R_diffuse _from the illumination site – was added to the linear-array data.

The NN prediction results were analyzed according to the percent deviation from the expected values as follows:

%Error=(μTrue−μEstimated)μTrue     (5)
 MathType@MTEF@5@5@+=feaafiart1ev1aaatCvAUfKttLearuWrP9MDH5MBPbIqV92AaeXatLxBI9gBaebbnrfifHhDYfgasaacH8akY=wiFfYdH8Gipec8Eeeu0xXdbba9frFj0=OqFfea0dXdd9vqai=hGuQ8kuc9pgc9s8qqaq=dirpe0xb9q8qiLsFr0=vr0=vr0dc8meaabaqaciaacaGaaeqabaqabeGadaaakeaacqGGLaqjcqWGfbqrcqWGYbGCcqWGYbGCcqWGVbWBcqWGYbGCcqGH9aqpdaWcaaqaamaabmGabaacciGae8hVd02aaSbaaSqaaiabdsfaujabdkhaYjabdwha1jabdwgaLbqabaGccqGHsislcqWF8oqBdaWgaaWcbaGaemyrauKaem4CamNaemiDaqNaemyAaKMaemyBa0MaemyyaeMaemiDaqNaemyzauMaemizaqgabeaaaOGaayjkaiaawMcaaaqaaiab=X7aTnaaBaaaleaacqWGubavcqWGYbGCcqWG1bqDcqWGLbqzaeqaaaaakiaaxMaacaWLjaWaaeWaceaacqaI1aqnaiaawIcacaGLPaaaaaa@582E@

%Error¯=∑i=1i=n|Errori|n     (6)
 MathType@MTEF@5@5@+=feaafiart1ev1aaatCvAUfKttLearuWrP9MDH5MBPbIqV92AaeXatLxBI9gBaebbnrfifHhDYfgasaacH8akY=wiFfYdH8Gipec8Eeeu0xXdbba9frFj0=OqFfea0dXdd9vqai=hGuQ8kuc9pgc9s8qqaq=dirpe0xb9q8qiLsFr0=vr0=vr0dc8meaabaqaciaacaGaaeqabaqabeGadaaakeaadaqdaaqaaiabcwcaLiabdweafjabdkhaYjabdkhaYjabd+gaVjabdkhaYbaacqGH9aqpdaWcaaqaamaaqahabaWaaqWaceaacqWGfbqrcqWGYbGCcqWGYbGCcqWGVbWBcqWGYbGCdaWgaaWcbaGaemyAaKgabeaaaOGaay5bSlaawIa7aaWcbaGaemyAaKMaeyypa0JaeGymaedabaGaemyAaKMaeyypa0JaemOBa4ganiabggHiLdaakeaacqWGUbGBaaGaaCzcaiaaxMaadaqadiqaaiabiAda2aGaayjkaiaawMcaaaaa@4F6D@

Equation (5) denotes the formula used to generate each of the error data points in the results graphs, whereas equation (6) was used to calculate the overall error for a set of data.

## Results

### Validation of calibrated system

**Figure 4:**
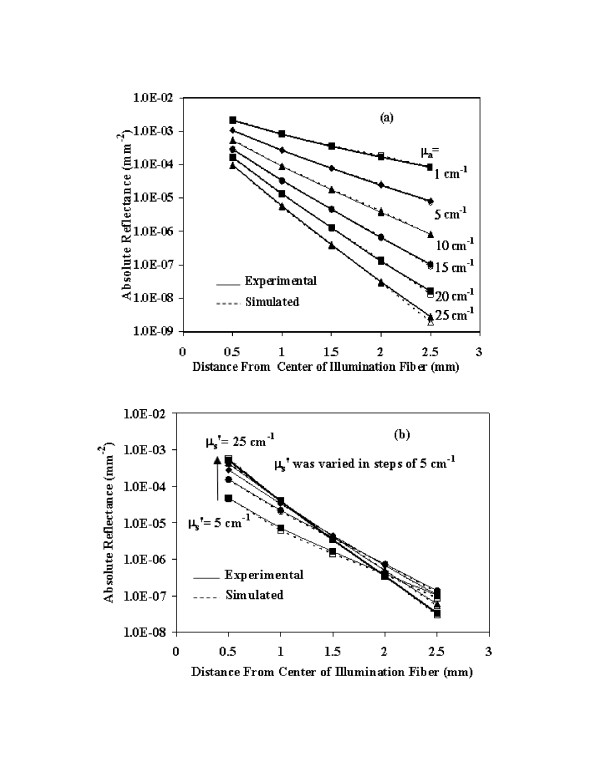
**Comparison of experimental and simulated reflectance with linear array probe**. These graphs present absolute diffuse reflectance data as determined by experimental measurements (filled shapes) and Monte Carlo simulations (open shapes). Graph (a) shows the effect of μ_a _on reflectance when μ′s
 MathType@MTEF@5@5@+=feaafiart1ev1aaatCvAUfKttLearuWrP9MDH5MBPbIqV92AaeXatLxBI9gBaebbnrfifHhDYfgasaacH8akY=wiFfYdH8Gipec8Eeeu0xXdbba9frFj0=OqFfea0dXdd9vqai=hGuQ8kuc9pgc9s8qqaq=dirpe0xb9q8qiLsFr0=vr0=vr0dc8meaabaqaciaacaGaaeqabaqabeGadaaakeaacuaH8oqBgaqbamaaBaaaleaacqqGZbWCaeqaaaaa@3007@ is held constant at 15 cm^-1^, whereas (b) shows the effect of μ_s _on the reflectance when μ_a _is held constant at 15 cm^-1^.

Initial validation of the fiberoptic based diffuse reflectance was carried out for the linear-array probe system. The calibration curves for converting CCD pixel counts to absolute intensity measurements were well-behaved, producing linear fits with R^2 ^values of 0.99 for all five detection fibers. Comparisons between experimental phantom measurements and Monte Carlo simulations for uniformly distributed optical property pairs (n = 10) indicated excellent agreement over a wide range of μ_a _and μ′s
 MathType@MTEF@5@5@+=feaafiart1ev1aaatCvAUfKttLearuWrP9MDH5MBPbIqV92AaeXatLxBI9gBaebbnrfifHhDYfgasaacH8akY=wiFfYdH8Gipec8Eeeu0xXdbba9frFj0=OqFfea0dXdd9vqai=hGuQ8kuc9pgc9s8qqaq=dirpe0xb9q8qiLsFr0=vr0=vr0dc8meaabaqaciaacaGaaeqabaqabeGadaaakeaacuaH8oqBgaqbamaaBaaaleaacqqGZbWCaeqaaaaa@3007@ values (Figure 4).

### Linear-array probe

**Figure 5:**
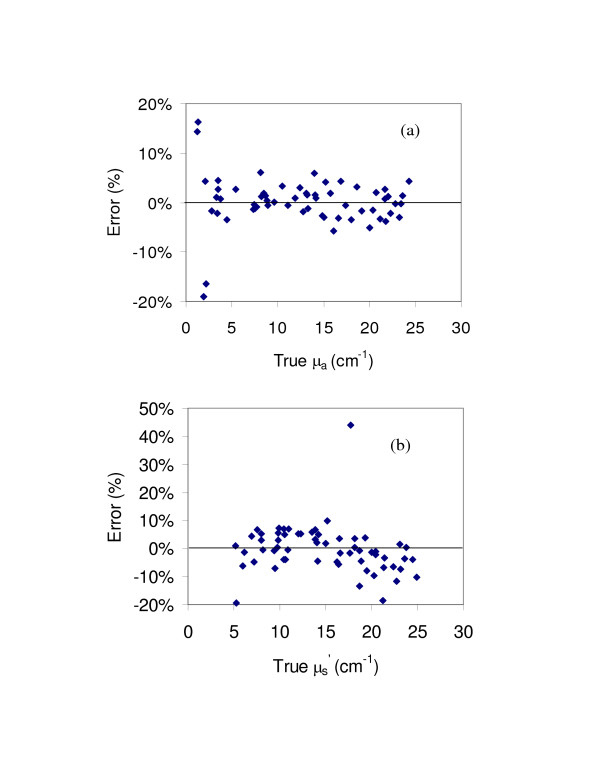
**Accuracy of optical property estimates with linear array probe**. These graphs present results for estimates of (a) μ_a _and (b) μ′s
 MathType@MTEF@5@5@+=feaafiart1ev1aaatCvAUfKttLearuWrP9MDH5MBPbIqV92AaeXatLxBI9gBaebbnrfifHhDYfgasaacH8akY=wiFfYdH8Gipec8Eeeu0xXdbba9frFj0=OqFfea0dXdd9vqai=hGuQ8kuc9pgc9s8qqaq=dirpe0xb9q8qiLsFr0=vr0=vr0dc8meaabaqaciaacaGaaeqabaqabeGadaaakeaacuaH8oqBgaqbamaaBaaaleaacqqGZbWCaeqaaaaa@3007@. Each of the 60 points represents the difference between predicted and true values.

A self-validation analysis was performed on simulated data to evaluate the performance and the theoretical limit of inverse model accuracy. The results of this analysis are shown in terms of mean prediction error in Table 1. Experimental evaluation was performed by measuring reflectance in 60 tissue phantoms with random optical properties over the following ranges: μ_a _from 1 to 25 cm^-1 ^and μ′s
 MathType@MTEF@5@5@+=feaafiart1ev1aaatCvAUfKttLearuWrP9MDH5MBPbIqV92AaeXatLxBI9gBaebbnrfifHhDYfgasaacH8akY=wiFfYdH8Gipec8Eeeu0xXdbba9frFj0=OqFfea0dXdd9vqai=hGuQ8kuc9pgc9s8qqaq=dirpe0xb9q8qiLsFr0=vr0=vr0dc8meaabaqaciaacaGaaeqabaqabeGadaaakeaacuaH8oqBgaqbamaaBaaaleaacqqGZbWCaeqaaaaa@3007@ from 5 to 25 cm^-1^. The optical property prediction accuracy for each measurement is displayed in Figure 5, with mean values displayed in Table 1. Low levels of error – less than 6% – are seen across most of the μ_a _range (3 cm^-1 ^up to 25 cm^-1^). However, in the μ_a _< 3 cm^-1 ^region, accuracy degraded rapidly, with four data points showing errors of 14–19%. This increased error is likely due to two factors: (a) the accuracy of the neural network routine degrades at the boundaries of the range over which it was calibrated and (b) since the μ_a _values in the aforementioned region are small, the same absolute levels of error that occur for larger μ_a _values result in much greater percentage errors. Figure 5b indicates that although the vast majority of μ′s
 MathType@MTEF@5@5@+=feaafiart1ev1aaatCvAUfKttLearuWrP9MDH5MBPbIqV92AaeXatLxBI9gBaebbnrfifHhDYfgasaacH8akY=wiFfYdH8Gipec8Eeeu0xXdbba9frFj0=OqFfea0dXdd9vqai=hGuQ8kuc9pgc9s8qqaq=dirpe0xb9q8qiLsFr0=vr0=vr0dc8meaabaqaciaacaGaaeqabaqabeGadaaakeaacuaH8oqBgaqbamaaBaaaleaacqqGZbWCaeqaaaaa@3007@ predictions were within ± 10% of the true value, a minor trend towards under-prediction at higher μ′s
 MathType@MTEF@5@5@+=feaafiart1ev1aaatCvAUfKttLearuWrP9MDH5MBPbIqV92AaeXatLxBI9gBaebbnrfifHhDYfgasaacH8akY=wiFfYdH8Gipec8Eeeu0xXdbba9frFj0=OqFfea0dXdd9vqai=hGuQ8kuc9pgc9s8qqaq=dirpe0xb9q8qiLsFr0=vr0=vr0dc8meaabaqaciaacaGaaeqabaqabeGadaaakeaacuaH8oqBgaqbamaaBaaaleaacqqGZbWCaeqaaaaa@3007@ values is seen. Two of the 20% under-predictions in this graph occurred for samples in which the true μ_a _value was low and prediction error for μ_a _was large. The data point showing the greatest error in μ′s
 MathType@MTEF@5@5@+=feaafiart1ev1aaatCvAUfKttLearuWrP9MDH5MBPbIqV92AaeXatLxBI9gBaebbnrfifHhDYfgasaacH8akY=wiFfYdH8Gipec8Eeeu0xXdbba9frFj0=OqFfea0dXdd9vqai=hGuQ8kuc9pgc9s8qqaq=dirpe0xb9q8qiLsFr0=vr0=vr0dc8meaabaqaciaacaGaaeqabaqabeGadaaakeaacuaH8oqBgaqbamaaBaaaleaacqqGZbWCaeqaaaaa@3007@ (43%), however, did not occur for a sample in which μ_a _error was high. One potential reason for this error may be air bubbles at the fiber tip which disrupt the fiber-sample coupling.

**Table 1:** Evaluation of predictive NN models for the linear-array probe system.

	E_simulated_ (n = 30)	E_measured_ (n = 60)
**Case A: **All Samples		
μ_a_	1.6%	3.2%
μ′s MathType@MTEF@5@5@+=feaafiart1ev1aaatCvAUfKttLearuWrP9MDH5MBPbIqV92AaeXatLxBI9gBaebbnrfifHhDYfgasaacH8akY=wiFfYdH8Gipec8Eeeu0xXdbba9frFj0=OqFfea0dXdd9vqai=hGuQ8kuc9pgc9s8qqaq=dirpe0xb9q8qiLsFr0=vr0=vr0dc8meaabaqaciaacaGaaeqabaqabeGadaaakeaacuaH8oqBgaqbamaaBaaaleaacqqGZbWCaeqaaaaa@3007@	0.9%	5.6%
**Case B: **Samples with μ_a _> 5 cm^-1^		
μ_a_	1.0%	2.2%
μ′s MathType@MTEF@5@5@+=feaafiart1ev1aaatCvAUfKttLearuWrP9MDH5MBPbIqV92AaeXatLxBI9gBaebbnrfifHhDYfgasaacH8akY=wiFfYdH8Gipec8Eeeu0xXdbba9frFj0=OqFfea0dXdd9vqai=hGuQ8kuc9pgc9s8qqaq=dirpe0xb9q8qiLsFr0=vr0=vr0dc8meaabaqaciaacaGaaeqabaqabeGadaaakeaacuaH8oqBgaqbamaaBaaaleaacqqGZbWCaeqaaaaa@3007@	1.0%	4.6%

Average errors (E) in prediction of absorption and reduced scattering coefficients are presented for two types of data sets: simulated (uniformly-distributed optical properties) and measured (randomly-distributed optical properties). Case A includes evaluation for the entire optical property range whereas for Case B, samples with μ_a _< 5 cm^-1 ^are omitted.

A summary of mean errors is presented in Table 1 for both the self-validation and experimental evaluation data. While the self-validation data set shows less error for μ′s
 MathType@MTEF@5@5@+=feaafiart1ev1aaatCvAUfKttLearuWrP9MDH5MBPbIqV92AaeXatLxBI9gBaebbnrfifHhDYfgasaacH8akY=wiFfYdH8Gipec8Eeeu0xXdbba9frFj0=OqFfea0dXdd9vqai=hGuQ8kuc9pgc9s8qqaq=dirpe0xb9q8qiLsFr0=vr0=vr0dc8meaabaqaciaacaGaaeqabaqabeGadaaakeaacuaH8oqBgaqbamaaBaaaleaacqqGZbWCaeqaaaaa@3007@ than for μ_a_, measured results indicate the opposite trend. The reason for this is addressed in the discussion section. In general, the self-validation results indicate significantly better estimations than the experimental results, due in part to unavoidable experimental errors (e.g., noise in the detected signal, nonuniformity of the sample, imperfect coupling, etc.). These small errors tend to be amplified for small μ_a _values, due to the two factors mentioned in the prior paragraph. Nevertheless, over the vast majority of the optical property range of interest, our approach provides a mean error level of less than 5%.

### Bifurcated probe and combined approach

**Figure 6:**
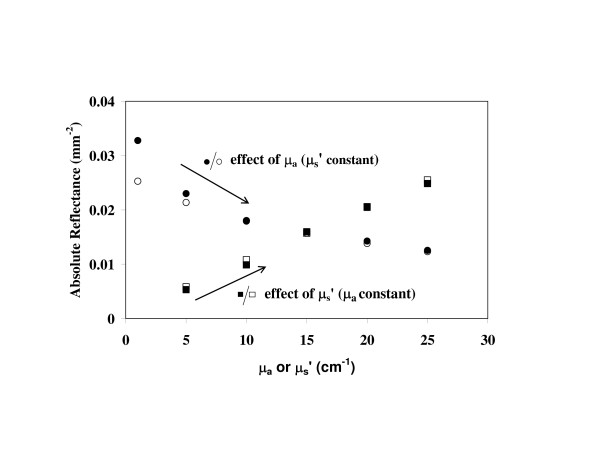
**Accuracy of diffuse reflectance measurements with bifurcated probe**. This graph provides a comparison of simulated (open shapes) and measured (filled shapes) diffuse reflectance for the bifurcated probe. Squares represent the effect of variations in μ′s
 MathType@MTEF@5@5@+=feaafiart1ev1aaatCvAUfKttLearuWrP9MDH5MBPbIqV92AaeXatLxBI9gBaebbnrfifHhDYfgasaacH8akY=wiFfYdH8Gipec8Eeeu0xXdbba9frFj0=OqFfea0dXdd9vqai=hGuQ8kuc9pgc9s8qqaq=dirpe0xb9q8qiLsFr0=vr0=vr0dc8meaabaqaciaacaGaaeqabaqabeGadaaakeaacuaH8oqBgaqbamaaBaaaleaacqqGZbWCaeqaaaaa@3007@ (for a constant μ_a _= 15 cm^-1^). Circles represent the effect of μ_a _(for a constant μ′s
 MathType@MTEF@5@5@+=feaafiart1ev1aaatCvAUfKttLearuWrP9MDH5MBPbIqV92AaeXatLxBI9gBaebbnrfifHhDYfgasaacH8akY=wiFfYdH8Gipec8Eeeu0xXdbba9frFj0=OqFfea0dXdd9vqai=hGuQ8kuc9pgc9s8qqaq=dirpe0xb9q8qiLsFr0=vr0=vr0dc8meaabaqaciaacaGaaeqabaqabeGadaaakeaacuaH8oqBgaqbamaaBaaaleaacqqGZbWCaeqaaaaa@3007@ = 15 cm^-1^).

Initial measurements were performed to assess the accuracy of bifurcated probe technique. A comparison of measured reflectance and simulated data for a range of μ_a _and μ′s
 MathType@MTEF@5@5@+=feaafiart1ev1aaatCvAUfKttLearuWrP9MDH5MBPbIqV92AaeXatLxBI9gBaebbnrfifHhDYfgasaacH8akY=wiFfYdH8Gipec8Eeeu0xXdbba9frFj0=OqFfea0dXdd9vqai=hGuQ8kuc9pgc9s8qqaq=dirpe0xb9q8qiLsFr0=vr0=vr0dc8meaabaqaciaacaGaaeqabaqabeGadaaakeaacuaH8oqBgaqbamaaBaaaleaacqqGZbWCaeqaaaaa@3007@ values is presented in Figure 6. Excellent agreement is observed between experimental and simulated data for most cases. However, a small deviation is seen at μ_a _= 5 cm^-1 ^and a large (25%) deviation is seen for μ_a _= 1 cm^-1^.

**Figure 7:**
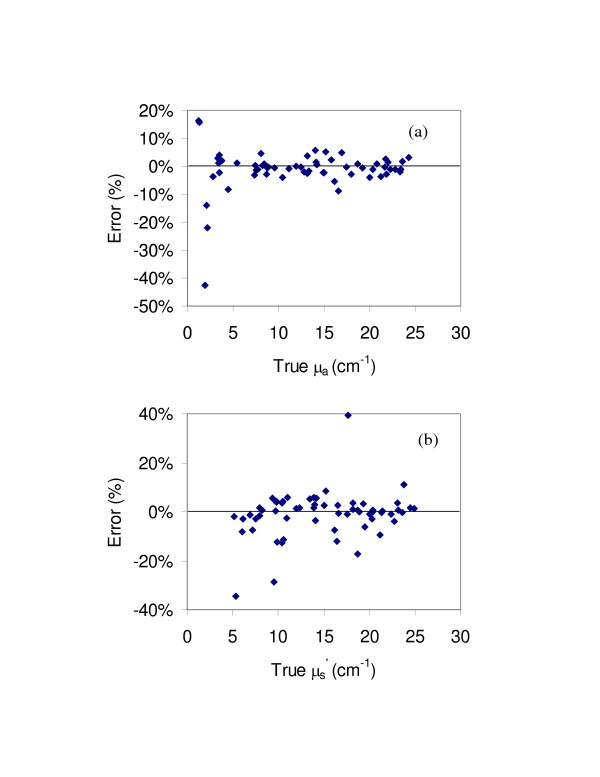
**Accuracy of optical property estimates with combined linear-bifurcated probe**. These graphs present results for estimates of (a) μ_a _and (b) μ′s
 MathType@MTEF@5@5@+=feaafiart1ev1aaatCvAUfKttLearuWrP9MDH5MBPbIqV92AaeXatLxBI9gBaebbnrfifHhDYfgasaacH8akY=wiFfYdH8Gipec8Eeeu0xXdbba9frFj0=OqFfea0dXdd9vqai=hGuQ8kuc9pgc9s8qqaq=dirpe0xb9q8qiLsFr0=vr0=vr0dc8meaabaqaciaacaGaaeqabaqabeGadaaakeaacuaH8oqBgaqbamaaBaaaleaacqqGZbWCaeqaaaaa@3007@. Each of the 60 data points represents the difference between predicted and true optical property values.

A predictive model was calibrated using simulated reflectance data corresponding to both linear array and bifurcated probe geometries. Self-validation analysis was performed using simulated data to evaluate the operation and theoretical limits of the model. The mean prediction errors for this case are presented in Table 2. The 60 tissue phantoms described in the prior section were then measured with the bifurcated probe and this data combined with the corresponding linear-array probe data. Prediction errors for this case are presented in Figure 7. The error graph for μ_a _shows all five samples with true values of 2.2 cm^-1 ^or less as being predicted with absolute errors of 14 to 42%. The model's diffuculty with low μ_a _samples also extended to μ′s
 MathType@MTEF@5@5@+=feaafiart1ev1aaatCvAUfKttLearuWrP9MDH5MBPbIqV92AaeXatLxBI9gBaebbnrfifHhDYfgasaacH8akY=wiFfYdH8Gipec8Eeeu0xXdbba9frFj0=OqFfea0dXdd9vqai=hGuQ8kuc9pgc9s8qqaq=dirpe0xb9q8qiLsFr0=vr0=vr0dc8meaabaqaciaacaGaaeqabaqabeGadaaakeaacuaH8oqBgaqbamaaBaaaleaacqqGZbWCaeqaaaaa@3007@ predictions, likely for the same reasons that large errors were produced at low optical property values for the linear array probe. Of the four data points showing the greatest μ′s
 MathType@MTEF@5@5@+=feaafiart1ev1aaatCvAUfKttLearuWrP9MDH5MBPbIqV92AaeXatLxBI9gBaebbnrfifHhDYfgasaacH8akY=wiFfYdH8Gipec8Eeeu0xXdbba9frFj0=OqFfea0dXdd9vqai=hGuQ8kuc9pgc9s8qqaq=dirpe0xb9q8qiLsFr0=vr0=vr0dc8meaabaqaciaacaGaaeqabaqabeGadaaakeaacuaH8oqBgaqbamaaBaaaleaacqqGZbWCaeqaaaaa@3007@ error levels, three corresponded to samples with μ_a _values under 2.2 cm^-1 ^that were poorly predicted by the model. The remaining value may be due to air bubbles as mentioned previously. Average percent errors (Table 2) for μ_a _and μ′s
 MathType@MTEF@5@5@+=feaafiart1ev1aaatCvAUfKttLearuWrP9MDH5MBPbIqV92AaeXatLxBI9gBaebbnrfifHhDYfgasaacH8akY=wiFfYdH8Gipec8Eeeu0xXdbba9frFj0=OqFfea0dXdd9vqai=hGuQ8kuc9pgc9s8qqaq=dirpe0xb9q8qiLsFr0=vr0=vr0dc8meaabaqaciaacaGaaeqabaqabeGadaaakeaacuaH8oqBgaqbamaaBaaaleaacqqGZbWCaeqaaaaa@3007@ were calculated for simulated as well as experimental data using both the complete set of samples and a set containing only samples with μ_a _values greater than 5 cm^-1^. When the entire data set was considered, the addition of the bifurcated probe data caused an increase in error for μ_a _while the μ′s
 MathType@MTEF@5@5@+=feaafiart1ev1aaatCvAUfKttLearuWrP9MDH5MBPbIqV92AaeXatLxBI9gBaebbnrfifHhDYfgasaacH8akY=wiFfYdH8Gipec8Eeeu0xXdbba9frFj0=OqFfea0dXdd9vqai=hGuQ8kuc9pgc9s8qqaq=dirpe0xb9q8qiLsFr0=vr0=vr0dc8meaabaqaciaacaGaaeqabaqabeGadaaakeaacuaH8oqBgaqbamaaBaaaleaacqqGZbWCaeqaaaaa@3007@ error level did not change appreciably. However, when the limited data set was analyzed, the change in geometry resulted in a 1% reduction in prediction error for μ′s
 MathType@MTEF@5@5@+=feaafiart1ev1aaatCvAUfKttLearuWrP9MDH5MBPbIqV92AaeXatLxBI9gBaebbnrfifHhDYfgasaacH8akY=wiFfYdH8Gipec8Eeeu0xXdbba9frFj0=OqFfea0dXdd9vqai=hGuQ8kuc9pgc9s8qqaq=dirpe0xb9q8qiLsFr0=vr0=vr0dc8meaabaqaciaacaGaaeqabaqabeGadaaakeaacuaH8oqBgaqbamaaBaaaleaacqqGZbWCaeqaaaaa@3007@ (a relative improvement of 24%) while the error for μ_a _remained unchanged.

**Table 2:** Evaluation of predictive NN models for the combined linear-array/bifurcated probe approach.

	E_simulated_ (n = 30)	E_measured_ (n = 60)
**Case A: **All samples		
μ_a_	2.1%	4.0%
μ′s MathType@MTEF@5@5@+=feaafiart1ev1aaatCvAUfKttLearuWrP9MDH5MBPbIqV92AaeXatLxBI9gBaebbnrfifHhDYfgasaacH8akY=wiFfYdH8Gipec8Eeeu0xXdbba9frFj0=OqFfea0dXdd9vqai=hGuQ8kuc9pgc9s8qqaq=dirpe0xb9q8qiLsFr0=vr0=vr0dc8meaabaqaciaacaGaaeqabaqabeGadaaakeaacuaH8oqBgaqbamaaBaaaleaacqqGZbWCaeqaaaaa@3007@	0.7%	5.5%
**Case B: **Samples with μ_a _> 5 cm^-1^		
μ_a_	0.9%	2.2%
μ′s MathType@MTEF@5@5@+=feaafiart1ev1aaatCvAUfKttLearuWrP9MDH5MBPbIqV92AaeXatLxBI9gBaebbnrfifHhDYfgasaacH8akY=wiFfYdH8Gipec8Eeeu0xXdbba9frFj0=OqFfea0dXdd9vqai=hGuQ8kuc9pgc9s8qqaq=dirpe0xb9q8qiLsFr0=vr0=vr0dc8meaabaqaciaacaGaaeqabaqabeGadaaakeaacuaH8oqBgaqbamaaBaaaleaacqqGZbWCaeqaaaaa@3007@	0.7%	3.7%

The cases and data sets in this table are the same as for Table 1.

## Discussion

### Evaluation of reflectance measurements

Initial comparisons between measured and simulated data helped to validate the basic system performance for the linear array (Figure 4) and bifurcated fiber (Figure 6) probes. These graphs indicate that good agreement was achieved between the experimentally measured diffuse reflectance and simulation results over the entire range of optical properties studied. For the linear array probe, experimental and modeled data showed small discrepancies at the most distant fiber and highest μ_a _values, as well as at lower μ′s
 MathType@MTEF@5@5@+=feaafiart1ev1aaatCvAUfKttLearuWrP9MDH5MBPbIqV92AaeXatLxBI9gBaebbnrfifHhDYfgasaacH8akY=wiFfYdH8Gipec8Eeeu0xXdbba9frFj0=OqFfea0dXdd9vqai=hGuQ8kuc9pgc9s8qqaq=dirpe0xb9q8qiLsFr0=vr0=vr0dc8meaabaqaciaacaGaaeqabaqabeGadaaakeaacuaH8oqBgaqbamaaBaaaleaacqqGZbWCaeqaaaaa@3007@ values. Bifurcated probe data show good agreement as well, with the exception of very low μ_a _values, where the experimental values are significantly larger than the simulation data. Further discussion of the source of this discrepancy is provided in the "Analysis of Combined Probe Approach" section.

### Notable trends in reflectance

Data in Figures 4 and 6 provide insight into general trends in reflectance as well as the performance of optical property estimation models. As seen in these figures and noted in our prior study [23], an increase in μ_a _causes relatively large decreases in reflectance, whereas increasing μ′s
 MathType@MTEF@5@5@+=feaafiart1ev1aaatCvAUfKttLearuWrP9MDH5MBPbIqV92AaeXatLxBI9gBaebbnrfifHhDYfgasaacH8akY=wiFfYdH8Gipec8Eeeu0xXdbba9frFj0=OqFfea0dXdd9vqai=hGuQ8kuc9pgc9s8qqaq=dirpe0xb9q8qiLsFr0=vr0=vr0dc8meaabaqaciaacaGaaeqabaqabeGadaaakeaacuaH8oqBgaqbamaaBaaaleaacqqGZbWCaeqaaaaa@3007@ produces less substantial variations. This is due to the fact that μ_a _directly affects absorption of photons all along the path from source to detector, whereas μ′s
 MathType@MTEF@5@5@+=feaafiart1ev1aaatCvAUfKttLearuWrP9MDH5MBPbIqV92AaeXatLxBI9gBaebbnrfifHhDYfgasaacH8akY=wiFfYdH8Gipec8Eeeu0xXdbba9frFj0=OqFfea0dXdd9vqai=hGuQ8kuc9pgc9s8qqaq=dirpe0xb9q8qiLsFr0=vr0=vr0dc8meaabaqaciaacaGaaeqabaqabeGadaaakeaacuaH8oqBgaqbamaaBaaaleaacqqGZbWCaeqaaaaa@3007@, influences the diffuse nature of photon propagation which has a less direct influence on attenuation. Given the larger changes in reflectance with μ_a _than μ′s
 MathType@MTEF@5@5@+=feaafiart1ev1aaatCvAUfKttLearuWrP9MDH5MBPbIqV92AaeXatLxBI9gBaebbnrfifHhDYfgasaacH8akY=wiFfYdH8Gipec8Eeeu0xXdbba9frFj0=OqFfea0dXdd9vqai=hGuQ8kuc9pgc9s8qqaq=dirpe0xb9q8qiLsFr0=vr0=vr0dc8meaabaqaciaacaGaaeqabaqabeGadaaakeaacuaH8oqBgaqbamaaBaaaleaacqqGZbWCaeqaaaaa@3007@, one would expect that for measurements with any significant level of error, the optical property estimation error would be greater for μ′s
 MathType@MTEF@5@5@+=feaafiart1ev1aaatCvAUfKttLearuWrP9MDH5MBPbIqV92AaeXatLxBI9gBaebbnrfifHhDYfgasaacH8akY=wiFfYdH8Gipec8Eeeu0xXdbba9frFj0=OqFfea0dXdd9vqai=hGuQ8kuc9pgc9s8qqaq=dirpe0xb9q8qiLsFr0=vr0=vr0dc8meaabaqaciaacaGaaeqabaqabeGadaaakeaacuaH8oqBgaqbamaaBaaaleaacqqGZbWCaeqaaaaa@3007@ than for μ_a_. This was shown to be true for evaluations with experimental data (Tables 1 and 2). While the self-validation results produced equivalent or greater errors for μ_a _than μ′s
 MathType@MTEF@5@5@+=feaafiart1ev1aaatCvAUfKttLearuWrP9MDH5MBPbIqV92AaeXatLxBI9gBaebbnrfifHhDYfgasaacH8akY=wiFfYdH8Gipec8Eeeu0xXdbba9frFj0=OqFfea0dXdd9vqai=hGuQ8kuc9pgc9s8qqaq=dirpe0xb9q8qiLsFr0=vr0=vr0dc8meaabaqaciaacaGaaeqabaqabeGadaaakeaacuaH8oqBgaqbamaaBaaaleaacqqGZbWCaeqaaaaa@3007@, these error levels were minimal. Figure 4a also indicates that the measured reflectance is slightly greater than the simulated reflectance for high μ′s
 MathType@MTEF@5@5@+=feaafiart1ev1aaatCvAUfKttLearuWrP9MDH5MBPbIqV92AaeXatLxBI9gBaebbnrfifHhDYfgasaacH8akY=wiFfYdH8Gipec8Eeeu0xXdbba9frFj0=OqFfea0dXdd9vqai=hGuQ8kuc9pgc9s8qqaq=dirpe0xb9q8qiLsFr0=vr0=vr0dc8meaabaqaciaacaGaaeqabaqabeGadaaakeaacuaH8oqBgaqbamaaBaaaleaacqqGZbWCaeqaaaaa@3007@ at the 2.5 mm position. These discrepancies appear to have been small enough to not significantly impact the predictions in Figure 5.

Reflectance data in Figure 4(b) indicate other interesting trends. Changes in reflectance intensity with μ′s
 MathType@MTEF@5@5@+=feaafiart1ev1aaatCvAUfKttLearuWrP9MDH5MBPbIqV92AaeXatLxBI9gBaebbnrfifHhDYfgasaacH8akY=wiFfYdH8Gipec8Eeeu0xXdbba9frFj0=OqFfea0dXdd9vqai=hGuQ8kuc9pgc9s8qqaq=dirpe0xb9q8qiLsFr0=vr0=vr0dc8meaabaqaciaacaGaaeqabaqabeGadaaakeaacuaH8oqBgaqbamaaBaaaleaacqqGZbWCaeqaaaaa@3007@ at the 0.5 mm fiber are significant when μ′s
 MathType@MTEF@5@5@+=feaafiart1ev1aaatCvAUfKttLearuWrP9MDH5MBPbIqV92AaeXatLxBI9gBaebbnrfifHhDYfgasaacH8akY=wiFfYdH8Gipec8Eeeu0xXdbba9frFj0=OqFfea0dXdd9vqai=hGuQ8kuc9pgc9s8qqaq=dirpe0xb9q8qiLsFr0=vr0=vr0dc8meaabaqaciaacaGaaeqabaqabeGadaaakeaacuaH8oqBgaqbamaaBaaaleaacqqGZbWCaeqaaaaa@3007@ values are small, but minimal when μ′s
 MathType@MTEF@5@5@+=feaafiart1ev1aaatCvAUfKttLearuWrP9MDH5MBPbIqV92AaeXatLxBI9gBaebbnrfifHhDYfgasaacH8akY=wiFfYdH8Gipec8Eeeu0xXdbba9frFj0=OqFfea0dXdd9vqai=hGuQ8kuc9pgc9s8qqaq=dirpe0xb9q8qiLsFr0=vr0=vr0dc8meaabaqaciaacaGaaeqabaqabeGadaaakeaacuaH8oqBgaqbamaaBaaaleaacqqGZbWCaeqaaaaa@3007@ is large. Conversely, at the 2.5 mm fiber, the change in reflectance as a function of μ′s
 MathType@MTEF@5@5@+=feaafiart1ev1aaatCvAUfKttLearuWrP9MDH5MBPbIqV92AaeXatLxBI9gBaebbnrfifHhDYfgasaacH8akY=wiFfYdH8Gipec8Eeeu0xXdbba9frFj0=OqFfea0dXdd9vqai=hGuQ8kuc9pgc9s8qqaq=dirpe0xb9q8qiLsFr0=vr0=vr0dc8meaabaqaciaacaGaaeqabaqabeGadaaakeaacuaH8oqBgaqbamaaBaaaleaacqqGZbWCaeqaaaaa@3007@ is small when μ′s
 MathType@MTEF@5@5@+=feaafiart1ev1aaatCvAUfKttLearuWrP9MDH5MBPbIqV92AaeXatLxBI9gBaebbnrfifHhDYfgasaacH8akY=wiFfYdH8Gipec8Eeeu0xXdbba9frFj0=OqFfea0dXdd9vqai=hGuQ8kuc9pgc9s8qqaq=dirpe0xb9q8qiLsFr0=vr0=vr0dc8meaabaqaciaacaGaaeqabaqabeGadaaakeaacuaH8oqBgaqbamaaBaaaleaacqqGZbWCaeqaaaaa@3007@ is low, and becomes larger at higher μ′s
 MathType@MTEF@5@5@+=feaafiart1ev1aaatCvAUfKttLearuWrP9MDH5MBPbIqV92AaeXatLxBI9gBaebbnrfifHhDYfgasaacH8akY=wiFfYdH8Gipec8Eeeu0xXdbba9frFj0=OqFfea0dXdd9vqai=hGuQ8kuc9pgc9s8qqaq=dirpe0xb9q8qiLsFr0=vr0=vr0dc8meaabaqaciaacaGaaeqabaqabeGadaaakeaacuaH8oqBgaqbamaaBaaaleaacqqGZbWCaeqaaaaa@3007@ values. Therefore, it is likely that reflectance from the 0.5 mm fiber is not very useful when μ′s
 MathType@MTEF@5@5@+=feaafiart1ev1aaatCvAUfKttLearuWrP9MDH5MBPbIqV92AaeXatLxBI9gBaebbnrfifHhDYfgasaacH8akY=wiFfYdH8Gipec8Eeeu0xXdbba9frFj0=OqFfea0dXdd9vqai=hGuQ8kuc9pgc9s8qqaq=dirpe0xb9q8qiLsFr0=vr0=vr0dc8meaabaqaciaacaGaaeqabaqabeGadaaakeaacuaH8oqBgaqbamaaBaaaleaacqqGZbWCaeqaaaaa@3007@ is high and reflectance from the 2.5 mm fiber is not very useful when μ′s
 MathType@MTEF@5@5@+=feaafiart1ev1aaatCvAUfKttLearuWrP9MDH5MBPbIqV92AaeXatLxBI9gBaebbnrfifHhDYfgasaacH8akY=wiFfYdH8Gipec8Eeeu0xXdbba9frFj0=OqFfea0dXdd9vqai=hGuQ8kuc9pgc9s8qqaq=dirpe0xb9q8qiLsFr0=vr0=vr0dc8meaabaqaciaacaGaaeqabaqabeGadaaakeaacuaH8oqBgaqbamaaBaaaleaacqqGZbWCaeqaaaaa@3007@ is low. Additionally, since reflectance at the 1.5 mm and 2 mm fibers does not appear to change significantly with μ′s
 MathType@MTEF@5@5@+=feaafiart1ev1aaatCvAUfKttLearuWrP9MDH5MBPbIqV92AaeXatLxBI9gBaebbnrfifHhDYfgasaacH8akY=wiFfYdH8Gipec8Eeeu0xXdbba9frFj0=OqFfea0dXdd9vqai=hGuQ8kuc9pgc9s8qqaq=dirpe0xb9q8qiLsFr0=vr0=vr0dc8meaabaqaciaacaGaaeqabaqabeGadaaakeaacuaH8oqBgaqbamaaBaaaleaacqqGZbWCaeqaaaaa@3007@, these values have minimal predictive value.

For the bifurcated probe geometry (Figure 6), increases in μ′s
 MathType@MTEF@5@5@+=feaafiart1ev1aaatCvAUfKttLearuWrP9MDH5MBPbIqV92AaeXatLxBI9gBaebbnrfifHhDYfgasaacH8akY=wiFfYdH8Gipec8Eeeu0xXdbba9frFj0=OqFfea0dXdd9vqai=hGuQ8kuc9pgc9s8qqaq=dirpe0xb9q8qiLsFr0=vr0=vr0dc8meaabaqaciaacaGaaeqabaqabeGadaaakeaacuaH8oqBgaqbamaaBaaaleaacqqGZbWCaeqaaaaa@3007@ produced an approximately linear increase in reflectance. As μ′s
 MathType@MTEF@5@5@+=feaafiart1ev1aaatCvAUfKttLearuWrP9MDH5MBPbIqV92AaeXatLxBI9gBaebbnrfifHhDYfgasaacH8akY=wiFfYdH8Gipec8Eeeu0xXdbba9frFj0=OqFfea0dXdd9vqai=hGuQ8kuc9pgc9s8qqaq=dirpe0xb9q8qiLsFr0=vr0=vr0dc8meaabaqaciaacaGaaeqabaqabeGadaaakeaacuaH8oqBgaqbamaaBaaaleaacqqGZbWCaeqaaaaa@3007@ was increased across the entire range from 5 to 25 cm^-1^, reflectance increased by 370% This early result formed the basis of our hypothesis that reflectance data at the point of illumination might provide useful information on μ′s
 MathType@MTEF@5@5@+=feaafiart1ev1aaatCvAUfKttLearuWrP9MDH5MBPbIqV92AaeXatLxBI9gBaebbnrfifHhDYfgasaacH8akY=wiFfYdH8Gipec8Eeeu0xXdbba9frFj0=OqFfea0dXdd9vqai=hGuQ8kuc9pgc9s8qqaq=dirpe0xb9q8qiLsFr0=vr0=vr0dc8meaabaqaciaacaGaaeqabaqabeGadaaakeaacuaH8oqBgaqbamaaBaaaleaacqqGZbWCaeqaaaaa@3007@ which could be measured via a combined bifurcated-linear probe geometry.

### Analysis of linear-array approach

The goal of this study was to develop a well-characterized second generation system that was highly accurate in the optical property range of interest. As mentioned previously, our prior system [23] was capable of estimating μ_a _and μ′s
 MathType@MTEF@5@5@+=feaafiart1ev1aaatCvAUfKttLearuWrP9MDH5MBPbIqV92AaeXatLxBI9gBaebbnrfifHhDYfgasaacH8akY=wiFfYdH8Gipec8Eeeu0xXdbba9frFj0=OqFfea0dXdd9vqai=hGuQ8kuc9pgc9s8qqaq=dirpe0xb9q8qiLsFr0=vr0=vr0dc8meaabaqaciaacaGaaeqabaqabeGadaaakeaacuaH8oqBgaqbamaaBaaaleaacqqGZbWCaeqaaaaa@3007@ with average errors of 12.5% and 16.2%, respectively. In the current study, the best results for the full optical property range was provided by the linear-array approach, which enabled prediction of μ_a _and μ′s
 MathType@MTEF@5@5@+=feaafiart1ev1aaatCvAUfKttLearuWrP9MDH5MBPbIqV92AaeXatLxBI9gBaebbnrfifHhDYfgasaacH8akY=wiFfYdH8Gipec8Eeeu0xXdbba9frFj0=OqFfea0dXdd9vqai=hGuQ8kuc9pgc9s8qqaq=dirpe0xb9q8qiLsFr0=vr0=vr0dc8meaabaqaciaacaGaaeqabaqabeGadaaakeaacuaH8oqBgaqbamaaBaaaleaacqqGZbWCaeqaaaaa@3007@ with errors of 3.2% and 5.6%, respectively. This represents reductions in error of 74% and 65% over our prior system. However, if the optical property range is restricted to include only μ_a _values above 5 cm^-1^, the combined approach provides even lower error levels: 2.2% and 3.7% for μ_a _and μ′s
 MathType@MTEF@5@5@+=feaafiart1ev1aaatCvAUfKttLearuWrP9MDH5MBPbIqV92AaeXatLxBI9gBaebbnrfifHhDYfgasaacH8akY=wiFfYdH8Gipec8Eeeu0xXdbba9frFj0=OqFfea0dXdd9vqai=hGuQ8kuc9pgc9s8qqaq=dirpe0xb9q8qiLsFr0=vr0=vr0dc8meaabaqaciaacaGaaeqabaqabeGadaaakeaacuaH8oqBgaqbamaaBaaaleaacqqGZbWCaeqaaaaa@3007@, respectively. These results represent a significant improvement over our prior system and provide evidence that the current approach has strong potential to provide accurate estimates of *in vivo *optical properties.

Exact comparisons of current results with prior studies are not possible due to a lack of data for diffuse-reflectance based systems in the optical property range of interest. However, our findings compare favorably with the limited data available in the literature. In one recent study [31], tissue phantoms with μ_a _values in the range of 1.3 cm^-1 ^to 31.8 cm^-1 ^were measured. The prediction error in this study ranged from 0.3% for a μ_a _of 14.4 cm^-1 ^to 14% for a μ_a _of 21.2 cm^-1^. A second study [32] found accuracy levels within 10% for μ_a _and 5% for μ′s
 MathType@MTEF@5@5@+=feaafiart1ev1aaatCvAUfKttLearuWrP9MDH5MBPbIqV92AaeXatLxBI9gBaebbnrfifHhDYfgasaacH8akY=wiFfYdH8Gipec8Eeeu0xXdbba9frFj0=OqFfea0dXdd9vqai=hGuQ8kuc9pgc9s8qqaq=dirpe0xb9q8qiLsFr0=vr0=vr0dc8meaabaqaciaacaGaaeqabaqabeGadaaakeaacuaH8oqBgaqbamaaBaaaleaacqqGZbWCaeqaaaaa@3007@ using tissue phantoms over an optical property range that included μ_a _values from 0.6 to 3.3 cm^-1 ^and μ′s
 MathType@MTEF@5@5@+=feaafiart1ev1aaatCvAUfKttLearuWrP9MDH5MBPbIqV92AaeXatLxBI9gBaebbnrfifHhDYfgasaacH8akY=wiFfYdH8Gipec8Eeeu0xXdbba9frFj0=OqFfea0dXdd9vqai=hGuQ8kuc9pgc9s8qqaq=dirpe0xb9q8qiLsFr0=vr0=vr0dc8meaabaqaciaacaGaaeqabaqabeGadaaakeaacuaH8oqBgaqbamaaBaaaleaacqqGZbWCaeqaaaaa@3007@ values from 10 to 22 cm^-1^. Not only are our current results comparable to those from prior studies in terms of prediction error, but the measurement of 60 random tissue phantoms enabled a more rigorous evaluation process than provided by prior studies.

### Analysis of combined probe approach

The linear-array geometry (Table 1) produced a higher level of prediction accuracy for μ_a _than for μ′s
 MathType@MTEF@5@5@+=feaafiart1ev1aaatCvAUfKttLearuWrP9MDH5MBPbIqV92AaeXatLxBI9gBaebbnrfifHhDYfgasaacH8akY=wiFfYdH8Gipec8Eeeu0xXdbba9frFj0=OqFfea0dXdd9vqai=hGuQ8kuc9pgc9s8qqaq=dirpe0xb9q8qiLsFr0=vr0=vr0dc8meaabaqaciaacaGaaeqabaqabeGadaaakeaacuaH8oqBgaqbamaaBaaaleaacqqGZbWCaeqaaaaa@3007@. Given the initial results from bifurcated probe measurements (Figure 6), it was expected that the combined linear-bifurcated approach would improve the accuracy of μ′s
 MathType@MTEF@5@5@+=feaafiart1ev1aaatCvAUfKttLearuWrP9MDH5MBPbIqV92AaeXatLxBI9gBaebbnrfifHhDYfgasaacH8akY=wiFfYdH8Gipec8Eeeu0xXdbba9frFj0=OqFfea0dXdd9vqai=hGuQ8kuc9pgc9s8qqaq=dirpe0xb9q8qiLsFr0=vr0=vr0dc8meaabaqaciaacaGaaeqabaqabeGadaaakeaacuaH8oqBgaqbamaaBaaaleaacqqGZbWCaeqaaaaa@3007@ predictions. Our findings confirm this hypothesis, although the improvements in accuracy are not obvious in all results. When all samples are considered, the implementation of the combined approach was seen to reduce the μ′s
 MathType@MTEF@5@5@+=feaafiart1ev1aaatCvAUfKttLearuWrP9MDH5MBPbIqV92AaeXatLxBI9gBaebbnrfifHhDYfgasaacH8akY=wiFfYdH8Gipec8Eeeu0xXdbba9frFj0=OqFfea0dXdd9vqai=hGuQ8kuc9pgc9s8qqaq=dirpe0xb9q8qiLsFr0=vr0=vr0dc8meaabaqaciaacaGaaeqabaqabeGadaaakeaacuaH8oqBgaqbamaaBaaaleaacqqGZbWCaeqaaaaa@3007@ estimation error from 5.6% to 5.5 %, while the error for μ_a _increased from 3.2 to 4.0%. When only samples with μ_a _> 5 cm^-1 ^were analyzed, μ′s
 MathType@MTEF@5@5@+=feaafiart1ev1aaatCvAUfKttLearuWrP9MDH5MBPbIqV92AaeXatLxBI9gBaebbnrfifHhDYfgasaacH8akY=wiFfYdH8Gipec8Eeeu0xXdbba9frFj0=OqFfea0dXdd9vqai=hGuQ8kuc9pgc9s8qqaq=dirpe0xb9q8qiLsFr0=vr0=vr0dc8meaabaqaciaacaGaaeqabaqabeGadaaakeaacuaH8oqBgaqbamaaBaaaleaacqqGZbWCaeqaaaaa@3007@ error improved from 4.6% to 3.7% (an improvement of 20%) while the μ_a _error remained constant at 2.2%. Therefore, in general, the implementation of the bifurcated approach produced an improvement in μ′s
 MathType@MTEF@5@5@+=feaafiart1ev1aaatCvAUfKttLearuWrP9MDH5MBPbIqV92AaeXatLxBI9gBaebbnrfifHhDYfgasaacH8akY=wiFfYdH8Gipec8Eeeu0xXdbba9frFj0=OqFfea0dXdd9vqai=hGuQ8kuc9pgc9s8qqaq=dirpe0xb9q8qiLsFr0=vr0=vr0dc8meaabaqaciaacaGaaeqabaqabeGadaaakeaacuaH8oqBgaqbamaaBaaaleaacqqGZbWCaeqaaaaa@3007@, possibly at the expense of a slight increase in μ_a _error.

The limited success of the bifurcated probe geometry and discrepancy between measured and simulated data at low μ_a _values (Figure 6) are likely related to two factors: (1) reflection off the probe face, which was simulated in our model as a perfect absorber and (2) discrepancies between the Henyey-Greenstein phase function used in the current study and the true phase function of the sample. While the probe face is not a perfect absorber, additional simulations performed with our model indicate that even if it were 100% reflective, the increase in detected signal for μ_a _= 1 cm^-1^, μ′s
 MathType@MTEF@5@5@+=feaafiart1ev1aaatCvAUfKttLearuWrP9MDH5MBPbIqV92AaeXatLxBI9gBaebbnrfifHhDYfgasaacH8akY=wiFfYdH8Gipec8Eeeu0xXdbba9frFj0=OqFfea0dXdd9vqai=hGuQ8kuc9pgc9s8qqaq=dirpe0xb9q8qiLsFr0=vr0=vr0dc8meaabaqaciaacaGaaeqabaqabeGadaaakeaacuaH8oqBgaqbamaaBaaaleaacqqGZbWCaeqaaaaa@3007@ = 15 cm^-1 ^would only be about 15%. Therefore, it is not likely that this is the primary for the discrepancy noted in Figure 6. Prior studies have indicated that for small source-detector separation distances, models based on the Henyey-Greenstein phase function may not produce accurate reflectance predictions due to underestimation of backscattered photons [33,34]. While neither of these prior studies included high μ_a _values, Mourant *et al*. provided simulation data for μ′s
 MathType@MTEF@5@5@+=feaafiart1ev1aaatCvAUfKttLearuWrP9MDH5MBPbIqV92AaeXatLxBI9gBaebbnrfifHhDYfgasaacH8akY=wiFfYdH8Gipec8Eeeu0xXdbba9frFj0=OqFfea0dXdd9vqai=hGuQ8kuc9pgc9s8qqaq=dirpe0xb9q8qiLsFr0=vr0=vr0dc8meaabaqaciaacaGaaeqabaqabeGadaaakeaacuaH8oqBgaqbamaaBaaaleaacqqGZbWCaeqaaaaa@3007@ = 12.2 cm^-1 ^and μ_a _= 0–2 cm^-1 ^in an adjacent fiber geometry which provides qualitative corroboration of the errors found with our bifurcated probe for μ_a_<5 cm^-1^. While the Mie phase function employed by Mourant *et al*. produces greater accuracy when simulating the behaviour of microspheres of a single diameter, the benefit of this approach may be reduced in complex biological tissue with a variety of scatterers. One of the only recent studies involving measurements of reflectance with a bifurcated probe showed minimal discrepancy between experimental results and Monte Carlo simulations employing a Henyey-Greenstein phase function [19]. Given these prior results, we believe that a bifurcated approach is valid at least for samples with μ_a _values of 5 cm^-1 ^or more. However, given the apparent lack of agreement in the literature there exists a need to further elucidate the true effect of phase function on reflectance measurements and in highly attenuating turbid media. Therefore, we intend perform a future study to thoroughly characterize the influence of phase function in highly attenuating samples and thus generate more definitive answers regarding the conditions (e.g., optical properties, probe geometries, scatterer sizes) for which Henyey-Greenstein and Mie phase functions are valid. Such research will further facilitate the simulation and development of reflectance systems that employ both single- and multi-fiber probes.

## Conclusion

This study represents a significant step towards the accomplishment of our long-term goal of performing *in vivo *measurements of tissue optical properties at UVA-VIS wavelengths. Revisions in instrumentation and procedures have improved our system's ability to measure optical properties in turbid media. A rigorous experimental evaluation of this system indicated decreases in average error of 74% and 65% for μ_a _and μ′s
 MathType@MTEF@5@5@+=feaafiart1ev1aaatCvAUfKttLearuWrP9MDH5MBPbIqV92AaeXatLxBI9gBaebbnrfifHhDYfgasaacH8akY=wiFfYdH8Gipec8Eeeu0xXdbba9frFj0=OqFfea0dXdd9vqai=hGuQ8kuc9pgc9s8qqaq=dirpe0xb9q8qiLsFr0=vr0=vr0dc8meaabaqaciaacaGaaeqabaqabeGadaaakeaacuaH8oqBgaqbamaaBaaaleaacqqGZbWCaeqaaaaa@3007@, respectively, in comparison with our prior study. Given that a mean optical property measurement error of less than 5% has been achieved, our approach has the potential to provide scientifically useful *in vivo *data. The inclusion of reflectance data from the illumination site produced a decrease of 24% in average error for predicting μ′s
 MathType@MTEF@5@5@+=feaafiart1ev1aaatCvAUfKttLearuWrP9MDH5MBPbIqV92AaeXatLxBI9gBaebbnrfifHhDYfgasaacH8akY=wiFfYdH8Gipec8Eeeu0xXdbba9frFj0=OqFfea0dXdd9vqai=hGuQ8kuc9pgc9s8qqaq=dirpe0xb9q8qiLsFr0=vr0=vr0dc8meaabaqaciaacaGaaeqabaqabeGadaaakeaacuaH8oqBgaqbamaaBaaaleaacqqGZbWCaeqaaaaa@3007@, yet the validity of this approach for samples with μ_a _values of about 5 cm^-1 ^or less is questionable and will require further research.

## Disclaimer

The opinions and conclusions stated in this paper are those of the authors and do not represent the official position of the U.S. Food and Drug Administration. The mention of commercial products, their sources, or their use in connection with material reported herein is not to be construed as either an actual or implied endorsement of such products by the U.S. Food and Drug Administration.

## Authors' contributions

DS calibrated the system, performed measurements of the phantoms, processed the reflectance data, and drafted the manuscript. AA assisted in modifying the experimental system and performing measurements as well as interpretation of the results. LSM designed, constructed and characterized the tissue phantoms. TJP conceived of and designed the study, constructed part of the system, developed the neural network model and helped to draft the manuscript. All authors read and approved the final manuscript.
